# Use of Sensory Analysis to Investigate the Influence of Climate Chambers and Other Process Variables in the Production of Sweet Wines

**DOI:** 10.3390/foods9040424

**Published:** 2020-04-03

**Authors:** M. Jesús Ruiz-Bejarano, Enrique Durán-Guerrero, Remedios Castro, Carmelo G. Barroso, M. Carmen Rodríguez-Dodero

**Affiliations:** Analytical Chemistry Department, Faculty of Sciences-IVAGRO, University of Cadiz, Agrifood Campus of International Excellence (ceiA3), Pol. Río San Pedro, s/n, Puerto Real, 11510 Cadiz, Spain; mariajesus.ruiz@uca.es (M.J.R.-B.); remedios.castro@uca.es (R.C.); carmelo.garcia@uca.es (C.G.B.); maricarmen.dodero@uca.es (M.C.R.-D.)

**Keywords:** sensory analysis, sweet wine, raisining, climate chamber

## Abstract

In this study, a climate chamber, as an alternative method, has been used to dry raisins and the sensory profiles of the sweet sherry wines obtained have been evaluated. Other important factors, namely grape variety, vintage, vinification conditions, as well as the ageing method and its length of time, have also been considered. When heavy rainfall had been registered, the musts extracted from grapes dried under controlled conditions in a climate chamber showed a lower intensity of the musty off-odor compared to those elaborated with sun-dried grapes. The wine fermented at low temperature with *Saccharomyces* *bayanus* scored the highest in citric and floral notes, and this was preferred over all the other wines that were evaluated. The wines aged in oak barrels were preferred to both, wines aged in the presence of oak chips as well as those aged without any wood contact. The use of climate chambers to dry the grapes that are going to be used for the elaboration of sweet wines appears to be an advantageous alternative to the traditional method, since it allows a more precise control of the process and highly valued sweet wines from a sensory point of view are obtained thereby.

## 1. Introduction

The production of sweet wines is commonly carried out with dehydrated grapes and there are three different processes to perform the water loss of the grapes: dehydration at a controlled temperature, humidity and ventilation; drying with non-controlled conditions and withering with eventual control of temperature and humidity and natural ventilation [1]. The sweet wines elaborated in Andalusia, Spain, are made from different white grape varieties, mainly Muscat and Pedro Ximénez, which are subjected to an ancient traditional method with non-controlled conditions: harvested grapes are dried in the open air, exposed to direct sunlight. This process is known as “asoleo” or sunning, where bunches of grapes are spread out on “redores”—esparto grass mats. During this sun-drying phase, which may take between seven and twenty days depending on the specific weather conditions [2,3], the bunches are turned over on a regular basis and covered at night. When the grapes are exposed to hours of intense sunshine, they gradually lose water and attain sugar concentration levels over 300 g/L; this process has an impact on their flavor profiles [4].

When this traditional procedure is applied, grapes may suffer undesired alterations that would have an impact on the quality of the final product; rainfalls particularly may contribute to the growth of certain fungi, which would lead to the loss of a considerable proportion of the treated grapes. In a worse scenario, highly toxic metabolites might appear in the wines that have been elaborated using such grapes [5,6,7,8]. For that reason, novel and more advanced techniques to be employed in the sweet wine industry should be sought.

There are several alternatives for a controlled dehydration of the grape, which would bring about a number of considerable advantages. On the one hand, withering methods with natural ventilation have been widely employed for the production of sweet wines in Italy, where the use of artificially heated air is not allowed [9,10,11,12,13,14]. On the other hand, climate chambers with forced convection of hot air have also been used for the raisining of grapes and other fruits [6,7,15,16,17,18,19,20]. Regardless of external weather conditions, both alternatives (with or without forced ventilation) could allow the adjustment of temperature and humidity over the drying process, thus reducing the length of time required for the desired raisining stage.

Research studies on Pedro Ximénez and Muscat sweet musts and wines made from grapes dried both under sunlight and within climate chambers with forced ventilation can be found in the literature [21,22,23,24,25,26], however, no studies have been found to comprise the subsequent optimization of the whole process, namely, fermentation conditions, ageing method and evolution of the product under wood contact.

With a view to proposing the use of climate chambers as an alternative to the traditional sun-drying method, we have evaluated the sensory profiles of sweet sherry wines elaborated from grapes dried in climatic chambers. Some important factors, such as grape variety, vintage, fermentation conditions, as well as the ageing method and time, have been considered in this study.

## 2. Materials and Methods

### 2.1. Production of the Wines

#### 2.1.1. Raisining

Two grape varieties (Muscat, M, and Pedro Ximénez, PX) from three consecutive vintages (V01, V02 and V03) were used for this research. The grapes were subjected to two different drying procedures: the traditional natural sun-drying method (T, either on a terrace in our research centre or at the vineyard) and controlled drying in a climate chamber (C).

For chamber drying, about 2000 kg of ripe grape bunches of each grape variety and vintage were collected from a local winery in the Jerez-Xérès-Sherry D.O. (Denomination of Origin) region. The grapes were dried in a climate chamber (Ibercex A.S.L., S.A., La Poveda, Spain) for about 5 days at 40 °C and 10% relative humidity. The bunches were uniformly distributed in a single layer inside the chamber. For the sun drying method, the grapes were spread out also in a single layer on “esparto” grass mats to dry under sunlight for about 10–15 days. They were turned over every day and covered at night. Grape weight loss was periodically monitored for both methods and the drying process was considered as completed when such weight loss reached about 35% of the original weight, at 20–21° Baume.

After this, grapes were separately destemmed, crushed and pressed by means of a vertical press (300 bars maximum). The initial pH of the must ranged between 3.6 and 3.8 and it was different for each experience, so it was adjusted to 3.5 by adding tartaric acid (Agrovin, Alcázar de San Juan, Spain). The concentration of total sulphur dioxide was also set at 120 mg/L by adding potassium metabisulfite (Agrovin).

#### 2.1.2. Fermentation

Five different conditions were tested for the partial fermentation of the musts obtained from vintage 01 Muscat grapes dried in the climate chamber (experiments E1 to E5, Table 1). In experiment E1, a *Saccharomyces cerevisiae* yeast inoculum (40 g/hL, Lalvin D254, Lallemand, Montreal, Canada) was employed. The fermentation was carried out at room temperature (less than 30 °C). In order to evaluate the effect of the addition of nitrogen, the conditions for experiment E2 were the same as for experiment E1 with the addition of some yeast nutrients (diammonium phosphate, 10 g/hL; Actimax Plus, Agrovin). For the evaluation of the employment of pellicular maceration, in experiment E3 the conditions were the same as for experiment E1 but the grapes were crushed without previous destemming and submitted to prefermentative pellicular maceration for 24 h at 4 °C using 3 g/hL of pectolytic enzymes (Enozym Arome, Agrovin), and then pressed. In order to check the influence of the type of yeast in the fermentation, the must from experiment E5 was fermented under the same conditions as for experiment E3 but employing *Saccharomyces bayanus* (40 g/hL, Uvaferm 43, Lallemand). Finally, to investigate the effect of the temperature in the fermentation, in experiment E4, the must was fermented at low temperature (less than 10 °C) with *S. bayanus*. Taking into account the recommendations from the supplier, this yeast was more suitable than *S. cerevisiae* to carry out the fermentation at lower temperatures.

The fermentations processes were carried out in duplicate. For vintages 02 and 03 the fermentation conditions were the same as those previously selected for vintage 01, that is, low temperature (about 10 °C) with *S. Bayanus* yeast. When the wine sugar content was around 90–100 g/L, alcohol up to 17–18° alc. was added to prevent any further fermentation. Alcohol content was determined by a distillation procedure and subsequent measurement of the density of the distillate.

#### 2.1.3. Ageing

After that, the selected Muscat wine from vintage 01 was subjected to ageing for 1 year using the following 3 parallel methods: For the first method, the wine was poured into 30 L medium toast American oak barrels (samples B); for the second method, the wine was put in contact with oak chips (Roblemor T, medium toast, Agrovin) at a dose of 4 g/L (samples identified by Ch) and for the third method, ageing took place without any contact with oak, in stainless steel tanks (samples S).

The sweet wines (Muscat and Pedro Ximénez) produced from vintages 02 and 03 were all aged in 30 L medium toasted American oak barrels (B). During this phase, all the wines were maintained in the same room at about 20 °C.

During each ageing phase, the samples were taken once every month over a whole year (S0 to S12). All of the samples were stored at 4 °C until their analysis.

### 2.2. Sensory Evaluation Methodology

The exclusively orthonasal sensory evaluation of the musts and wines was carried out in duplicate by a panel of between 13 and 17 members depending on the test. The samples were not tasted with the mouth, so the retronasal sensory evaluation was not performed. All the panel members had a medium level of experience in sensory analysis of sweet wines [27]. In addition, in order to validate the reproducibility of the judges’ assessment, the descriptive profiles of 2 reference samples were obtained; for each descriptor, a two-factor ANOVA (judges × samples) of the descriptive data was performed to determine the consistency of the assessments. The tasting sessions were held in a standard tasting room equipped with separate booths [28] at 22 °C.

Quantitative descriptive analysis [29], and in some cases triangle discrimination [30] or ranking tests [31], were performed.

Twenty-milliliter samples were presented in blue glasses (typically used for olive oil sensory analysis [32]), so that color would not influence the panel members’ assessments. The sample containers were topped with a watch-glass to minimize any possible aroma losses. The samples were identified by numerical codes composed by three random figures, and different for each judge. For triangle discrimination, samples were presented in order to let appear each possible disposition of the samples an equal number of times, but for descriptive analysis and ranking tests a randomized presentation was employed.

After performing the triangle test, the judges were also asked to provide a quantitative assessment of the detected differences. The following 5-point scale was used to rate such differences: not present (0), low (1), medium (2), strong (3) and very strong (4).

In respect to descriptive profile tests, as a preliminary stage to determine the appropriate descriptors that would better define the samples, a representative number of must and wine samples from the whole set were presented to the judges for them to provide qualitative descriptions for each one. The descriptors with a mention frequency over 5 were selected; i.e., fruity, citric, ripe fruit, raisin, floral, honey, herbaceous, vinous, lactic, musty, chemical character and oak. The final worksheet also included aromatic intensity and olfactory quality as descriptors. Each descriptor was scored according to a nine-point scale (0: absent; 2: light; 4: medium; 6: intense; 8: very intense), and also the olfactory quality of each sample was evaluated according to a structured nine-point scale (0: bad; 2: mediocre; 4: acceptable; 6: good; 8: very good) [33]. Table 2 presents the definitions agreed by all the judges for each descriptor as well as the standard used to recognize and quantify intensity (8) for each one of them. Both citric and floral were connected to a Muscat grape distillate as a standard because these were the two main descriptors of this cultivar. Therefore, the same standard could be perfectly used for the recognition of both descriptors.

### 2.3. Statistical Analysis

The statistical analyses were carried out using Statistica 8.0 (StatSoft GmbH, Hamburg, Germany), and Microsoft Office Excel 2010 applications.

The treatment of the data from the triangle test was based on the tabulated statistic data [30] with respect to difference tests, by setting the α-error at 0.05. Friedman test was applied for the treatment of the data from the olfactory quality ranking, as specified by the corresponding ISO standard [31].

An analysis of variance (ANOVA) was performed on the quantitative sensory data from the descriptive assessments. However, an acceptable dispersion of sensory data could complicate the discrimination between groups of samples for a particular descriptor (especially when professional or very trained panels are not employed). Therefore, it is proposed the subsequent application of multivariate statistical techniques that take into account the whole group of differences and similarities for all variables.

A Principal Component Analysis (PCA) was performed in order to highlighting the similarity of the samples, and to determine the main contributors to any of the differences found between them. Missing data were replaced by the average value of that variable in the group. The minimum eigenvalue to select a principal component was set at 1.0, and a factor rotation according to varimax normalized method was applied to confirm a correlation with the PCA. Loadings greater than 0.7 identified those variables well correlated to PCs.

In order to identify those descriptors that better differentiate between clusters of samples, a Linear Discriminant Analysis (LDA) was performed according to Wilks’ lambda statistic, and the so-called forward stepwise method was employed. According to this methodology, the discrimination model is built step-by-step by reviewing all the variables at each step and evaluating which one contributes the most to the discrimination between clusters. The F-to-enter and F-to-remove were set at 1 and 0.

## 3. Results and Discussion

### 3.1. Effect of the Raisining Method on the Aroma of Raisin Musts

The musts obtained after traditional drying of the grapes from the first year (V01) were compared with those dried in a climatic chamber by means of a triangle test (*N* = 13 judges, α = 0.05). In the case of Muscat, 10 out of the 13 judges identified the different sample, while in the discrimination of Pedro Ximénez musts, 8 judges detected the different sample. Given that according to the standard, 8 is the minimum number of coincidental judgments required to confirm a significant difference between the samples, we could conclude that the Muscat musts and also those of Pedro Ximénez presented a significantly different aromatic profile if the grapes had been raisined in the sun or in a climate chamber. The differences were valued by the judges as being of a moderate intensity (1.7 ± 0.4 on a 0–4 scale).

An ANOVA was then applied to the scores obtained from the descriptive test on these V01 musts with the objective of identifying the aromatic notes that were responsible for the differences that had previously been confirmed by the triangle test. The resulting data are shown in Table 3. Of all the positive notes considered, only fruity and raisins could statistically differentiate (*p* < 0.05) the musts from Muscat grapes treated by the 2 drying methods tested, being higher the scores for the musts obtained under controlled conditions. From Pedro Ximénez musts, the results were similar, although the differences were not significant. Having said that, the most interesting result is that those musts obtained from grapes dried in chambers were perceived as having a lower intensity in the olfactory fungal defect (musty). The detection of such a defect in the musts from either a drying method, seems to indicate that the grapes had been previously contaminated, probably due to the rainfalls during the days before that year’s harvesting period. As a result, the musts of grapes dried in climate chambers were better evaluated (aromatic quality).

When the data from the second vintage (V02) musts were analyzed, it was found that non-significant differences between the average scores for some of the defects in the Muscat musts could justify a better olfactory rating when their grapes had been dried in a climate chamber. However, this preference did not reach statistical significance. On the other hand, no differences could be confirmed for V03 musts that had been dried either in a climate chamber or by means of the traditional sun-drying method.

It should be taken into account that the grapes from these vintages (V02, and particularly in V03) arrived at the pilot plant in a better sanitary condition. This could explain their similar evolution regardless of the raisining method employed, as opposed to what happened with V01 grapes, which was characterized by intense rainfalls on the days prior to their harvest. Other authors neither found significant sensory differences between musts obtained by grapes dried traditionally or in a controlled way [34].

Due to the intrinsic variance of sensory data, it is often difficult to confirm differences between samples based on the results obtained from an analysis of variance. Therefore, in order to evaluate the minor differences between each of these descriptors that may come into sight when comparing “asoleo” and climate chamber musts, a PCA was applied to the sensory scores of all the must samples from V01, V02 and V03. The PCA allowed determining four principal components that account for 86.7% of the total variance. The loads (data not shown) confirmed a relationship between PC1 (40.1% of the variance explained) and the drying method, given its correlation with olfactory descriptors such as ripe fruit and raisins; and PC2 (22.6% of the variance explained) that represents a greater aromatic complexity, as it correlates with aromatic intensity and citric and floral notes, which contribute to better aromatic quality. On the other hand, PC3 and PC4 (with 16.1% and 7.9% of the explained variance, respectively) are related to aromatic defects (lactic, musty, herbaceous to PC3 and chemical to PC4).

**Figure 1:**
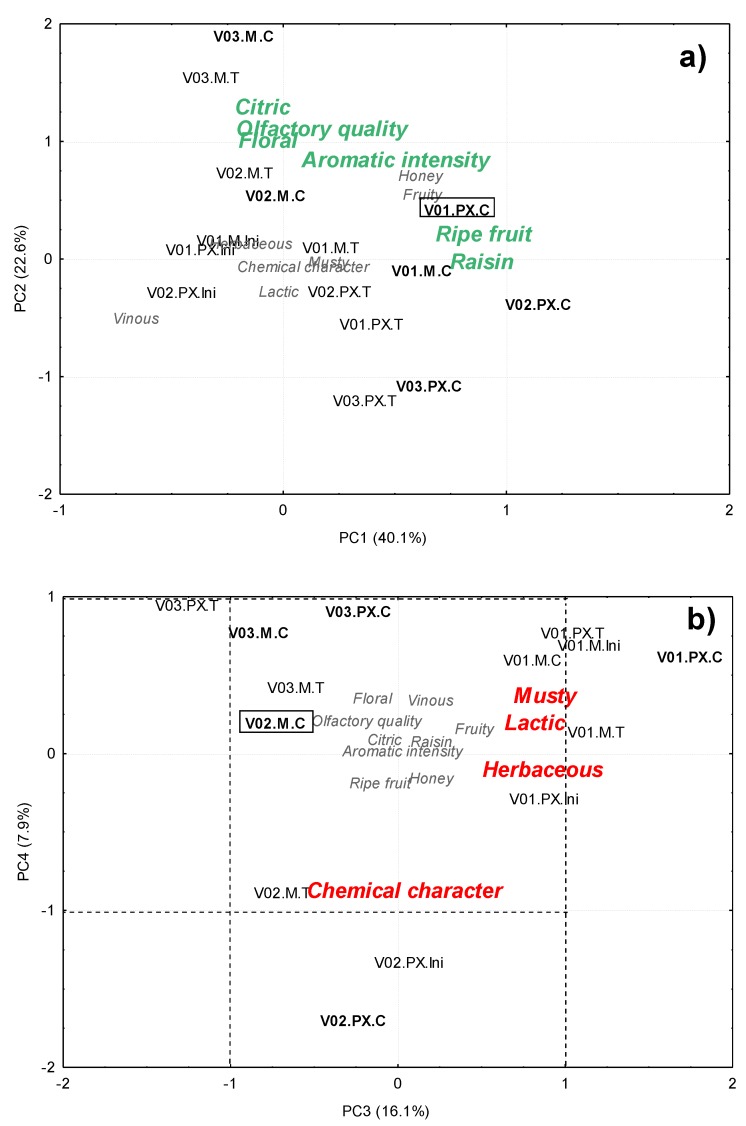
Principal components analysis of the sensory scores of musts from raisins obtained by the two different drying methods tested. Representation of both, the samples scores and the principal component loadings, onto the new space defined by: (**a**) PC1 and PC2 and (**b**) PC3 and PC4. The names of the samples indicate the vintage.variety.raisining method.

When the sensory profiles were transferred to this new representation, it can be seen that for PC1 (Figure 1a), the raisin musts from the climatic chamber (C, in bold type) were located to the right of their corresponding raisins obtained by means of the sun-drying method (T). Muscat musts were positioned at the highest PC2 values, which means that their aroma was perceived as having a greater complexity, since they exhibited a greater intensity in highly appreciated citric and floral notes. It is well known that the sensory profile of musts depends on their variety [35,36]. Interestingly, the only Pedro Ximénez must with high PC2 values was the one made from V01 grapes dried in a climate chamber (V01.PX.C).

On the other hand, PC3 and PC4 allowed us to differentiate the musts according to their harvest (Figure 1b). As can be seen, the musts from harvest 03 (V03) were located in the most distant area from the defects (lactic, musty, herbaceous and chemical character), with no influence from the variety or the raisining method. This would confirm the important influence of the vintage on the quality of raisin musts, as some authors had already pointed out in previous studies [37,38].

In any case, the most interesting result from our research is that the Muscat grape musts from the 02 vintage (V02.M.C), whose grapes were treated in a climate chamber, were also located in this defect-free area. It is therefore confirmed that the use of a climate chamber for the raisining phase of the grapes avoided some of the risks associated to adverse weather conditions, by allowing the hygienic drying of the grapes and preventing the appearance of fungi and its associated aromatic defects, while food safety was also preserved. In addition, in some cases the musts would exhibit a greater aromatic complexity. It should also be added that the drying time was reduced by 50–66%. At this point, it could be concluded that the capacity of the sensory analysis as a methodology to control the hygienic and sanitary state of musts was demonstrated.

### 3.2. Selecting the Fermenting Conditions for Sweet Wines Made from Grapes Dried in a Climate Chamber

The following step was the evaluation of some fermenting conditions in the production of sweet wine such as the type of yeast, the addition of nitrogen, the employment of pellicular maceration or the fermentation temperature (Table 1). The wines obtained were compared with each other by means of a ranking test according to olfactory quality. Thus, since the threshold of statistical significance (α = 0.05) for the 17-member panel that participated in the test was 9.5, and that the experimental value reached 9.8, it could then be confirmed that some differences between the wines in terms of olfactory quality were perceived. The outcome of the ranking test was E4^1^ (63) > E1^1^ (56) > E3^1,2^ (55) > E5^2^ (43) > E2^2^ (36), where the different superscripts indicate that these wines were perceived differently with regards to their olfactory qualities. The wine that had been fermented at low temperature with *S. bayanus* (E4) was the best rated, although the difference with the wine fermented with *S. cerevisiae* at room temperature (E1) was not significant. The nitrogen-added wine was granted the lowest score (E2), which agreed with the conclusions by other authors [39]. Finally, the wines subjected to pellicular maceration (E3 and E5) presented intermediate ratings, although the one fermented employing *S. cerevisiae* (E3) was slightly better scored.

The wines were also evaluated by means of descriptive tests. Table 4 shows the results from the ANOVA, according to which, the floral and citric notes have the greatest power of discrimination (lowest *p*-values) between the wines produced. These are attributed the highest score in the ranking test for wine E4 (fermented with *S. bayanus* at low temperature), which was characterized by high scores in Muscat typical *citric* and *floral* notes (in agreement with the observations of other authors regarding this yeast [40]), while the wine with the lowest score (E2, with added nitrogen) was characterized by very low intensities in these positive notes, as well as a clear olfactory defect of a chemical nature. Likewise, the fruity and floral notes of wines E3 and E5 (both subjected to pellicular maceration with pectolytic enzymes), were high. This was in agreement with the results observed by other authors regarding the volatile composition and sensory profile of Muscat wines employing this type of enzymes [41,42]. Comparing the obtained evaluation for these two wines (E3 and E5) obtained under the same conditions, but with different yeast strains, slight differences can be observed, so the effect of the employed yeast seems to be not so relevant, unless the temperature of fermentation is low (E4).

Consequently, the E4 test was selected as the preferred fermenting conditions for the production of sweet wines from grapes dried in a climate chamber, namely with *Saccharomyces bayanus* yeast, at a controlled temperature not greater than 10 °C, no nitrogen added and non-pellicular maceration in the presence of pectolytic enzymes. The above fermenting conditions were used for the rest of the research.

### 3.3. Selecting the Ageing Method for Sweet Wines made from Grapes Dried in a Climate Chamber

**Figure 2:**
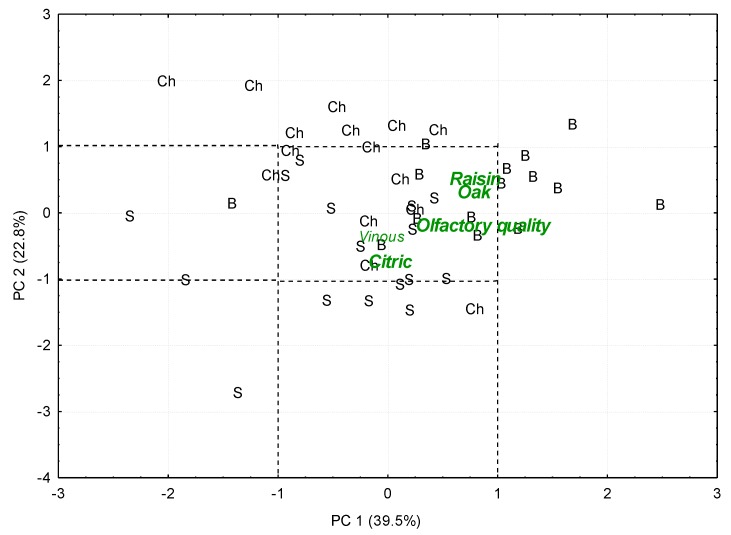
Principal components analysis of the sensory data of the same Muscat wine, vintage 01, aged under 3 different ageing methods (Ch: oak chips; B: oak barrels and S: stainless steel containers). Representation of both, the samples scores and the principal component loadings, onto the plane defined by the first two PCs.

The Muscat wine from V01 fermented under the preferred conditions (E4) was subjected to ageing for 1 year using oak chips (Ch), oak barrels (B) and stainless steel containers (S). A LDA where the ageing method was taken as the cluster variable was applied to the sensory profiles of the sampled wines. This would allow the identification of the descriptors that best differentiate them. Since a high percentage of the wines (86.9%) were correctly classified, a PCA was performed on such most significant descriptors (i.e., those with *p* < 0.05): oak, olfactory quality, vinous, raisin, citric, musty, honey, fruity and ripe fruit. Figure 2 shows PC1 and PC2, which explain respectively 39.5% and 22.8% of the initial variance. Where PC1 is correlated with the descriptors raisin and oak, and also with the olfactory quality, while PC2 is inversely related to the intensity of the citric notes.

When the wines are represented on this same plane, it can be seen that those wines, which were aged without any contact with oak (S) presented the lowest values for PC2, that is to say, they exhibited high intensity levels of citric notes, a typical character of Muscat wines when kept under these ageing conditions. Other authors [43] already confirmed that the degree to which these typical varietal attributes (or the compounds responsible for them) were maintained throughout the whole process from grape to the final wine depends on how the fermentation and ageing were conducted.

On the other hand, wines aged in oak barrels (B) were found on the right area of the graph (high values for PC1), which implies high intensity in oak notes and a very clearly perceived raisin note, both contributing to a highly valued olfactory quality.

With regard to wines aged in contact with oak chips (Ch), their position on the plane indicates low aromatic intensities of positive character notes and therefore it did not appear to be the best ageing system in principle.

This clear differentiation regarding ageing time corresponded to the discrimination observed for the same samples regarding the volatile composition [26]. Thus, the results obtained by means of sensory analysis seemed to confirm again the benefits of traditional ageing in oak casks as a method for ageing sweet wines made from raisins produced in a climate chamber.

### 3.4. Effect of the Length of The Ageing Period on the Aromatic Profile of Sweet Wines Made from Grapes Dried in a Climate Chamber

The previously selected vinification (E4) and ageing (B) methods were applied to the Muscat and Pedro Ximénez musts obtained from the V02 and V03 grapes dried in climate chambers, and the ageing process was monitored for 1 year. In the case of V01 grapes, only the wines from the Muscat variety were aged, since those from the Pedro Ximénez variety were accidentally contaminated by external causes at the beginning of the process and became unsuitable for this study.

**Figure 3:**
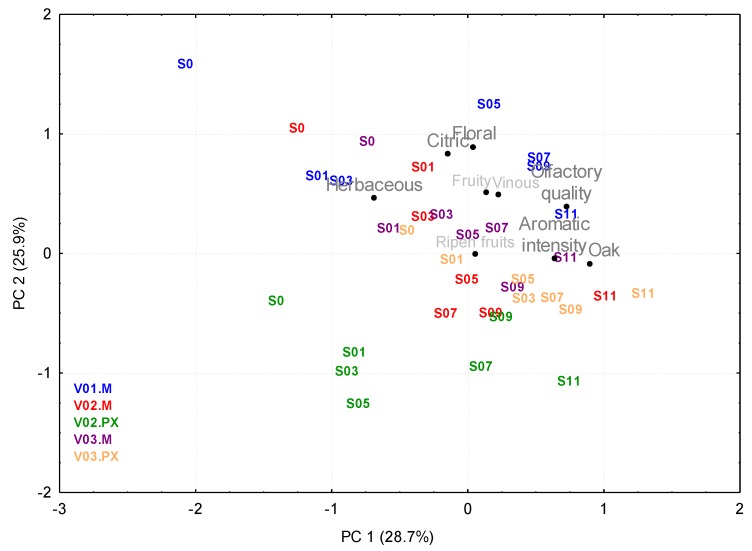
Principal components analysis of the sensory data of PX and Muscat wines from vintages V01, V02 and V03 fermented under the same conditions (E4) and aged for one year in oak barrels. Representation of both, the samples scores and the principal component loadings, onto the plane defined by the first two PCs.

In order to identify the variables that best discriminate wines according to their ageing time length, a LDA was performed on the initial (S0), intermediate (S6) and final (S12) samples, where the ageing time would be the cluster variable. The descriptors with the highest discrimination capacity were: aromatic intensity, fruity, citric, ripe fruit, floral, herbaceous, oak, vinous and together with olfactory quality (data not shown). The exclusive use of these sensory descriptors minimizes the amount of unnecessary data and is expected to improve the estimate and interpretation of the new components found by the PCA analysis, which resulted in three PCs (explaining 74% of the variance). Since standardized coefficients greater than 0.7 were considered as significant, it could be confirmed that the descriptors aromatic intensity, oak and olfactory quality correlated well with PC1 (which explained 28.7% of the variance), as well as herbaceous, although this latter correlated inversely, since it was a negative coefficient. With the same criterion, PC2 (explains 25.9% of the variance) correlated well with the citric and floral notes; while PC3 (19.4% of the variance explained) did so with the fruity and ripe fruit notes. Figure 3 represents the wines on the PC1–PC2 plane, although, for a clearer display, only the wines without any ageing (S0) and the samples from ageing wines that had been taken every other month (S01, S03, S05, S07, S09 and S11) are shown. As above mentioned, according to their loads, PC2 seems to be related to a greater aromatic complexity characterized by clear citric and floral notes, which would explain the higher values of this component in wines produced from Muscat grapes, with characteristic and intense primary aromas that are then enhanced during the fermentation phase (and which are located at the upper part of the graph), as opposed to the more neutral PX (at the lower area). It also seems logical that the scores for this PC decreased as the ageing time increases (from S0 to S11), given that the varietal typicity was partly eclipsed by the appearance of tertiary aromas.

It can be seen from the graph that for each vintage and variety the wines were sorted according to their ageing time along PC1 (from S0 to S11, from left to right). According to the loads of this component, the arrangement of the samples implies greater intensities of oak and aromatic intensity as the ageing process progresses, and what is more interesting, the perceived olfactory quality improved. The scores increased significantly over the first month, a behavior that has already been described by other authors [44] in relation to the volatile compounds found in sweet sherry wines during their oxidative ageing. It can also be seen that, based on the very different scores of the initial wines from the three vintages considered—which can be related to the different qualities of the musts as confirmed in previous sections—the wines that completed the ageing process presented similar PC1 values. This would attribute a certain buffering effect to the ageing process, contrary to what other authors concluded [45], who observed that different grape varieties have different wood extraction capacities, and that the ageing in wood barrels may enhance intrinsic varietal aroma differences.

It could be concluded that with regards to wood ageing and from the sensory point of view, ageing in oak barrels improved the olfactory perception of all the sweet wines made from grapes dried in a climate chamber, whether it be Muscat or Pedro Ximénez. Nevertheless, the effect became more noteworthy when the grapes were from less optimal vintages from the aromatic point of view.

## 4. Conclusions

In this study, sensory analysis was proven to be a reliable methodology to evaluate different process variables in the production of sweet wines, in which climate chambers to dehydrate the grapes were employed. The wine fermented at low temperature with *Saccharomyces bayanus* scored the highest in citric and floral notes, and this was preferred over all the other wines that were evaluated. Regarding the ageing stage, the wines aged in oak barrels were preferred to both, wines aged in the presence of oak chips as well as those aged without any wood contact. In addition, the use of climate chambers is a valuable alternative technique for the dehydration of grapes, compared to the traditional sun-drying method, because they allow a more precise control on the process and facilitate the solution to some production problems such as the musty off-odors associated to fungus contamination.

## Figures and Tables

**Table 1:** Experimental conditions of the 5 fermentation assays carried out with Muscat must extracted from grapes dried in a climatic chamber in vintage 01.

Assay	Yeast	Nitrogen	Pellicular Maceration with Pectolytic Enzymes	Fermentation Temperature
**E1**	*S. cerevisiae*	No	No	Room
**E2**	*S. cerevisiae*	Yes	No	Room
**E3**	*S. cerevisiae*	No	Yes	Room
**E4**	*S. bayanus*	No	No	Low
**E5**	*S. bayanus*	No	Yes	Room

**Table 2:** Aroma descriptors used for sample assessment.

Descriptor	Definition	Standard
Aromatic intensity	Intensity of overall olfactory perception by the orthonasal route	Commercial Muscat
Fruity	Raw material (grapes)	Muscat and PX grapes
Citric	Reminiscence of the common note in citrus fruits	Muscat grape distillate, 30% alc. (*v*/*v*)
Ripe fruit	Reminiscence of white stone fruit in an advanced state of ripeness	Hydroalcoholic solution 30% alc. (*v*/*v*) with pieces of ripe apple and pear
Raisin	Reminiscence of the dried raw material	Raisins Muscat and PX
Floral	Common note in flowers, whatever the species	Muscat grape distillate, 30% alc. (*v*/*v*)
Honey	Reminiscence of honey	Flower honey
Herbaceous	Sharp green note	Commercial herbaceous pomace
Vinous	Reminiscence of recently fermented sherry wine	Freshly fermented sherry wine (and frozen until tasting)
Lactic	Characteristic note of wines that undergo malolactic fermentation	White wine that has recently undergone lactic fermentation (and frozen until tasting)
Musty	Olfactory defect with earthy or mushroomy notes	Hydroalcoholic mixture 15% alc. (*v*/*v*) with addition of mushroomy extract from Le nez du Vin *
Chemical character	Olfactory defect with notes of solvent, glue or medicinal	Hydroalcoholic mixture 15% alc. (*v*/*v*) with addition of aromatic glue and medicinal extracts from Le nez du Vin *
Oak	Reminiscence of American oak wood	Hydroalcoholic solution 15% alc. (*v*/*v*) with American oak chips
Olfactory quality	Overall measurement by the orthonasal route of aromatic complexity and intensity and the absence of defects	Commercial Muscat and PX wines

* Le nez du Vin, Jean Lenoir Ed., 2006.

**Table 3:** ANOVA of sensory scores of musts, vintages 01–03. Values are expressed as average ± standard deviation. Differences are significant at *p* < 0.05.

Descriptor	Muscat V01			Pedro Ximénez V01			Muscat V02			Pedro Ximénez V02			Muscat V03			Pedro Ximénez V03		
	Sun-Drying	Climate Chamber	*p*	Sun-Drying	Climate Chamber	*p*	Sun-Drying	Climate Chamber	*p*	Sun-Drying	Climate Chamber	*p*	Sun-Drying	Climate Chamber	*p*	Sun- Drying	Climate Chamber	*p*
Aromatic intensity Fruity Citric Ripe fruit Raisin Floral Honey Herbaceous Vinous Lactic Musty Chemical character Olfactory quality	4.5 ± 1.4 3.9 ± 1.2 1.1 ± 1.4 3.4 ± 2.2 3.3 ± 1.2 2.0 ± 1.9 1.2 ± 1.6 1.9 ± 1.4 0.9 ± 1.7 0.4 ± 0.9 1.9 ± 2.2 0.1 ± 0.5 2.8 ± 1.9	4.2 ± 1.3 4.7 ± 0.8 0.9 ± 1.3 2.6 ± 2.0 4.5 ± 1.5 1.7 ± 1.6 2.3 ± 2.5 1.2 ± 1.9 1.2 ± 1.9 0.5 ± 1.1 1.3 ± 1.8 0.2 ± 0.5 3.6 ± 1.7	0.152 0.045 0.215 0.276 0.049 0.557 0.345 0.219 0.337 0.216 0.071 0.125 0.115	4.5 ± 1.4 4.0 ± 1.5 1.2 ± 1.6 3.3 ± 1.8 2.0 ± 1.6 1.3 ± 1.6 1.8 ± 2.4 1.6 ± 0.6 1.2 ± 2.0 0.5 ± 0.8 3.2 ± 1.8 0.3 ± 0.5 2.5 ± 2.3	4.3 ± 1.0 5.5 ± 2.1 0.7 ± 1.0 3.0 ± 1.8 2.3 ± 1.7 2.2 ± 2.1 2.3 ± 1.9 1.7 ± 0.7 0.3 ± 0.8 0.0 ± 0.0 1.2 ± 0.9 0.0 ± 0.0 4.7 ± 0.8	0.817 0.079 0.535 0.876 0.096 0.516 0.701 0.305 0.375 0.174 0.061 0.144 0.051	3.9 ± 1.2 3.9 ± 0.7 2.2 ± 0.9 2.1 ± 1.2 1.1 ± 0.3 2.1 ± 0.4 1.1 ± 0.3 1.0 ± 0.7 0.3 ± 0.6 0.1 ± 0.5 0.6 ± 0.4 0.4 ± 0.5 2.8 ± 1.1	4.3 ± 1.4 4.2 ± 1.1 2.3 ± 0.7 2.4 ± 0.9 1.9 ± 0.7 2.7 ± 0.2 1.6 ± 1.3 0.7 ± 0.4 0.2 ± 0.5 0.1 ± 0.6 0.1 ± 0.3 0.2 ± 0.5 5.1 ± 1.2	0.223 0.251 0.655 0.811 0.339 0.158 0.729 0.922 0.976 0.873 0.234 0.287 0.088	4.2 ± 0.9 2.9 ± 0.8 0.7 ± 0.4 3.0 ± 1.1 3.4 ± 1.0 0.9 ± 0.5 1.9 ± 0.6 0.7 ± 0.4 0.9 ± 0.7 0.3 ± 0.6 0.2 ± 0.3 1.2 ± 0.5 3.7 ± 0.4	4.3 ± 1.2 3.5 ± 1.0 0.6 ± 0.2 3.4 ± 0.8 2.7 ± 1.2 0.9 ± 0.6 1.3 ± 1.1 1.0 ± 0.4 0.7 ± 1.1 0.2 ± 0.8 0.2 ± 0.3 1.0 ± 0.5 3.9 ± 0.2	0.463 0.407 0.845 0.310 0.976 0.617 0.363 0.669 0.721 0.683 0.886 0.997 0.134	6.0 ± 0.9 4.1 ± 1.1 3.0 ± 0.8 2.5 ± 0.7 2.5 ± 1.0 8.1 ± 1.7 2.1 ± 0.7 0.5 ± 0.6 0.3 ± 0.5 0.0 ± 0.0 0.0 ± 0.0 0.0 ± 0.0 7.1 ± 1.1	4.5 ± 1.2 4.2 ± 0.8 2.5 ± 0.6 2.5 ± 0.3 1.0 ± 0.9 5.2 ± 2.1 2.4 ± 0.8 0.4 ± 0.5 0.2 ± 0.6 0.0 ± 0.0 0.0 ± 0.0 0.0 ± 0.0 6.4 ± 1.3	0.276 0.533 0.753 0.194 0.893 0.750 0.537 0.722 0.690 0.972 0.859 0.989 0.402	3.0 ± 0.6 1.5 ± 0.8 0.5 ± 0.4 2.5 ± 0.8 3.0 ± 0.6 0.6 ± 0.6 0.2 ± 0.4 0.5 ± 0.7 1.2 ± 1.1 0.2 ± 0.5 0.0 ± 0.0 0.0 ± 0.0 4.1 ± 0.9	4.5 ± 0.9 2.0 ± 0.7 0.4 ± 0.4 3.0 ± 0.7 2.9 ± 1.1 1.1 ± 0.5 0.4 ± 0.7 1.0 ± 0.8 1.5 ± 0.5 0.5 ± 0.6 0.0 ± 0.0 0.0 ± 0.0 4.5 ± 0.6	0.465 0.937 0.207 0.359 0.788 0.263 0.417 0.675 0.287 0.866 0.512 0.644 0.230

**Table 4:** ANOVA of sensory scores of wines for vintage 01 made under different fermentation conditions, as described in the text. Mean values and standard deviations are shown.

Descriptor	E1	E2	E3	E4	E5	*p*
**Aromatic intensity** **Fruity** **Citric** * Ripe fruits **Raisins** **Floral** * Honey **Herbaceous** **Vinous** **Lactic** **Musty** **Chemical character** * Olfactory quality *	4.1 ± 0.5 4.2 ± 0.3 2.2 ± 0.7 ^2^ 1.9 ± 0.4 1.9 ± 0.5 3.6 ± 0.8 ^1,2^ 0.9 ± 0.2 1.1 ± 0.4 2.5 ± 0.5 0.2 ± 0.3 0.6 ± 0.3 0.8 ± 0.3 ^1^ 4.4 ± 0.4 ^3^	4.2 ± 0.6 2.7 ± 0.4 1.4 ± 0.5 ^1^ 1.5 ± 0.6 1.6 ± 0.5 3.0 ± 0.7 ^1^ 0.8 ± 0.4 2.1 ± 0.6 2.3 ± 0.7 0.5 ± 0.3 1.1 ± 0.4 2.1 ± 0.3 ^2^ 2.5 ± 0.6 ^1^	4.4 ± 0.6 3.8 ± 0.3 2.8 ± 0.3 ^2,3^ 2.1 ± 0.4 2.2 ± 0.4 3.9 ± 0.5 ^1,2^ 1.5 ± 0.5 1.3 ± 0.6 2.5 ± 0.5 0.5 ± 0.3 1.1 ± 0.6 0.6 ± 0.3 ^1^ 3.1 ± 0.5 ^1,2^	4.1 ± 0.5 4.1 ± 0.4 3.1 ± 0.4 ^3^ 1.7 ± 0.5 2.3 ± 0.4 4.1 ± 0.6 ^2^ 1.2 ± 0.3 1.4 ± 0.5 2.9 ± 0.6 0.5 ± 0.3 0.9 ± 0.4 1.0 ± 0.3 ^1^ 4.5 ± 0.5 ^3^	4.2 ± 0.5 3.8 ± 0.5 2.7 ± 0.3 ^2,3^ 1.4 ± 0.4 1.1 ± 0.3 4.0 ± 0.8 ^2^ 1.1 ± 0.5 9 ± 0.7 2.8 ± 0.6 0.5 ± 0.4 1.5 ± 0.5 0.8 ± 0.2 ^1^ 3.8 ± 0.5 ^2,3^	0.97 0.11 0.04 0.87 0.84 0.07 0.54 0.83 0.87 0.99 0.86 0.04 0.05

* Values are statistically significant at *p* < 0.05. Different superscript numbers indicate that tasters perceived a significant different intensity.
